# Plant Studies May Lead Us to Rethink the Concept of Behavior

**DOI:** 10.3389/fpsyg.2016.00622

**Published:** 2016-04-28

**Authors:** Fatima Cvrčková, Viktor Žárský, Anton Markoš

**Affiliations:** ^1^Department of Experimental Plant Biology, Faculty of Science, Charles University in PraguePrague, Czech Republic; ^2^Department of Philosophy and History of Science, Faculty of Science, Charles University in PraguePrague, Czech Republic

**Keywords:** behavior, operative definition, developmental plasticity, development, physiology, movement

David Stenhouse's classical definition of intelligence as “*adaptively variable behavior within the lifetime of the individual*” (Stenhouse, 1974, in Trewavas, 2003), widely accepted among biologists, explicitly refers to behavior, requiring thus a previous understanding of this concept. Here, we are providing a biologists' perspective, which may differ from that of psychologists, economists or cognitive scientists, in hope that we may prompt colleagues from those fields to participate in quest for a common language.

## Behavior is not defined consistently

It is a common misconception that rigorous scientific study is impossible without an unambiguous definition of its subject, although the definition may evolve together with the field of research. A good definition ought to be operational (i.e., providing criteria to discern entities that fit it), essential (i.e., only including necessary and sufficient attributes), widely applicable and succinct (Levitis et al., 2009). Nevertheless, whole scientific fields continue to flourish without a consensual definition of their subject. Biology is a prime example: Barbieri (2003) lists over 60 definitions of life from the scientific literature, about a third of them quite recent. Similarly, cognitive sciences apparently can live without a clear-cut formal definition of cognition (Abramson, 2013; Whissell et al., 2013), and the same holds for the definition of behavior. Within the field of psychology, a wide consensus is as far nowadays as it was nearly a century ago (Robinson, 1918, Abramson and Place, 2005), and the situation is not better in biology. Levitis et al. (2009) collected about 25 different operational definitions of behavior from biological literature. In a survey among biologists engaged in behavioral research, they then asked the respondents to decide on a collection of general statements describing candidate essential features of behavior, and to determine which specific examples of biological phenomena constitute behavior. Somewhat disconcertingly, even professional scholars often based their views on intuitive understanding rather than on formal criteria, and sometimes expressed internally inconsistent opinions.

Unlike in e.g., mathematics, the absence of formal definitions does not seriously hamper fruitful research in empirical natural sciences such as biology, as long as some consensual intuitive understanding of the central concepts exists. This apparently is the case for life, cognition, and behavior, concepts borrowed from everyday speech and hence endowed with a broad semantic field (compare Robinson, 1918; Markoš and Cvrčková, 2013). Insisting on formal definitions may even get in the way of progress if these happen to (unwillingly) exclude areas of interest that otherwise might be relevant. Unfortunately, exactly this may be now happening in respect to the concept of “behavior.”

## The uneasy relationship between behavior and development

Many attempts to define behavior are rooted in animal biology and psychology, emphasizing a clear demarcation line between behavior and development. The latter is generally acknowledged merely as one of many important factors shaping behavior, together with evolutionary, hereditary and environmental influences (e.g., Bertossa, 2011; Crews et al., 2015), leading to an *exclusive* “development of behavior” perspective. Hence the proposed consensus definition of behavior as “*the internally coordinated responses (actions or inactions) of whole living organisms (individuals or groups) to internal and/or external stimuli, excluding responses more easily understood as developmental changes*” (Levitis et al., 2009). This view is widely shared (e.g., Bertossa, 2011) and is easily understandable in the context of animal ontogeny with its body plans and more or less rigorously set developmental pathways. However, no such sharp line is considered between behavioral and physiological processes; indeed, a strict separation of these fields would disqualify, e.g., I.P. Pavlov's classical conditioning experiments with drooling dogs (reviewed in Jarius and Wildermann, 2015).

An implicit feature of behavior is present in many definitions, and in nearly all of the examples in the above-cited study (Levitis et al., 2009), though not always overtly: behavior is generally assumed to involve active (and, at best, rapid) movement in the physical space. Consequently, most of the behavioral responses studied in psychology and ethology are linked to locomotion, which does not occur in plants. Many scholars (including some plant biologists) thus easily agree that behavior is not a useful concept for describing what plants do. This brings memories of an important lesson from the history. A similar tacit assumption concerning sex, which was also considered to be inseparable from locomotion, together with a non-reflected belief in a fundamental difference between animals and the rest of the living world (traceable to Aristotle), has hampered acceptance of the idea that plants are sexual organisms. Many prominent scholars denied plant sexuality up to the beginning of the 19th century, long after its initial description by A. Zalužanský at the end of the 16th century and by the better known J. Camerarius a century later (see Žárský and Tupý, 1995; Funk, 2013). We suggest that similar non-reflected assumptions may contribute to the problem of defining behavior in a manner which would not severally restrict the field of phenomena observable in plants that can (and should) be studied from the behavioral perspective.

We believe that in plants development, physiological responses, and behavior always go hand in hand. Plant behavior in the sense of the above-cited exclusive definition would be limited merely to rare instances of organ movements as in *Mimosa pudica* (reviewed in Abramson and Chicas-Mosier, 2016), a somewhat marginal topic for the plant biologists engaged in the nascent fields of “plant intelligence studies” (e.g., Trewavas, 2003; Meyer et al., 2014; Trewavas, 2014; van Loon, 2016, for additional references see Calvo and Baluška, 2015) or “plant behavioral ecology” (Cahill and McNickle, 2011; Gianoli, 2015). As sessile organisms whose cells are enclosed in relatively rigid cell walls, plants move through the physical space, slowly but surely, by growing—through extending existing organs and generating new ones, often in response to environmental cues. Plant post-embryonic ontogeny follows a species-specific algorithm rather than a body plan, based on plastic use of repetitive modules, incessantly shaped by the environment and by individual experience. Thus, plant biologists understand behavior as encompassing developmental plasticity within the limits of a broad norm of reaction (Crews et al., 2015) or phenotypic space (Pigliucci, 2010), in an *inclusive* “development as behavior” perspective (Trewavas, 2009). Such a position, generally compatible e.g., with the current theoretical framework of the Developmental Systems Theory (Oyama, 2000), or with the notion of ontogeny as a manifestation of species-specific culture (Markoš, 2002), can be traced back to Darwin's classical work on the growth and movement of root tips (Darwin and Darwin, 1880), and allows characterizing processes such as navigation of roots across obstacles, including other roots (Falik et al., 2005; Depuydt, 2014), non-genetic individual variability of seed dormancy breaking (Silvertown, 1984, but see Wagmann et al., 2012 for a critical re-evaluation of the extent of such variability) or sprouting of dormant buds (Thellier et al., 2004), in terms of behavioral biology (e.g., Ciszak et al., 2012). Yet, in the above-cited survey (Levitis et al., 2009), only two of the examples to be judged as “behavior or something else” involved plants (photonastic movement of mature leaves and closure of stomata), and the respondents thus had no opportunity to decide whether developmental plasticity should be interpreted as behavior.

## An attempt at an inclusive operational definition

We are thus dealing with two mutually incompatible views of behavior: (i) an exclusive one, represented by the working definition proposed by Levitis et al. (2009), possibly well suited for animal studies but obviously too restrictive for plants, and (ii) an inclusive one, which may become problematic in animal studies. Reducing the working definition of behavior to “*internally coordinated responses (actions or inactions) of whole living organisms (individuals or groups) to internal and/or external stimuli*” would not solve our problem, since it would classify every developmental change as behavior. Behavioral biologists are unlikely to make such a concession. Unless we accept separate lineage-specific definitions for animals and plants, a new definition should encompass a consensus between animal-oriented and plant-oriented biologists. Levitis et al. (2009) found such a consensus both overall and within each group (as opposed to an overall majority consensus, biased by the majority of animal biologists in the poll) only for the following three statements:

“*A developmental change is usually not a behavior.*” In the light of plant biology tradition this is somewhat puzzling, hinting at the need to examine what is understood as developmental change by animal and plant biologists. Growth and production of vegetative organs may not be perceived as a “change” by plant biologists, because it is just something plants do all the time. Moreover, the magic word “usually” leaves some interpretation freedom. Perhaps this statement should be rephrased to distinguish between developmental processes following a strict, genetically determined plan, such as early animal embryogenesis, or development of a single flower, and those involving decisions made within broader limits permitted by a species-specific developmental algorithm. The latter, in our opinion, should be admitted as behavior.
“*Behavior is always influenced by the internal processes of the individual.*”
“*A behavior is always in response to a stimulus or set of stimuli, but the stimulus can be either internal or external.*” While the last two statements do not present any obvious problem, it is worth noting that internal processes and stimuli also include memory traces of past experience (see Cvrčková et al., 2009).

Remarkably, a marked disagreement between animal and plant biologists was found concerning two proposed features of behavior in the Levitis et al. (2009) study. Only plant biologists were willing to admit the status of behavior to phenomena comprising only parts of individuals, which is understandable given the problematic nature of individuality in plants (Clarke, 2012). Because animal behavior is commonly accepted as occurring at the level of both individuals and groups (such as flocks of birds or swarms of insects), we propose to substitute “*living entities*,” understood as automomous agents (Kauffman, 2000), instead of “*whole living organisms (individuals or groups)*.” This would also legitimize the existing use of the concept of behavior in cell-level studies (e.g., Tokoyoda et al., 2004; Marrone et al., 2011; Hodgkinson et al., 2014, and many others). On the other hand, animal biologists surprisingly often disagreed with the statement that behavior must be directly observable, recordable, and measurable, supported by a majority of plant biologists (Levitis et al., 2009). It is, however, not clear whether the disagreement was caused by the requirement for observability or (more likely) by the word “directly.” Neither of these two features should therefore be central to our proposed definition.

Taking into account the considerations discussed above, we propose defining behavior as “*observable consequences of the choices a living entity makes in response to external or internal stimuli.*” We emphasize that the word “choice” is used here in the sense of adopting one of at least two alternative fates, or trajectories, in the state-space available to the living being in question, including, but not limited to, movement (or lack thereof) in the physical space. By no means does the use of this word imply involvement of a mind or consciousness. In our sense, flipping a metabolically or genetically wired switch also is a choice if its probability depends on outside stimuli and internal settings (previous history) of the biological system concerned.

## Time to cross some borders?

Nevertheless, a new definition may not be enough, unless accompanied by a certain shift of perspective. Carefully mapping the trench separating behavioral phenomena from those “*more easily understood as developmental changes*” (Levitis et al., 2009) would, in turn, in the interests of consistency call for a similar demarcation between behavioral biology and physiology, a task which may be impossible to attain without discarding many classical behavioral studies. Instead, we propose that the existing overlap between behavioral science and physiology ought to be taken as a glorious example of peaceful co-existence of two disciplines addressing the same subject from two different angles. Such a view, based on delimiting the fields of inquiry by methodology and perspective rather than by subject, is extensible also to the relationship between behavioral sciences and developmental biology. We believe that the recent plant investigations provide a sufficient justification for doing so.

## Author contributions

FC drafted the manuscript and performed the final editing. VŽ and AM contributed ideas and parts of the text, and participated in editing the manuscript. All authors have approved the final version prior to submission.

## Funding

This work has been supported by the Ministry of Education, Youth, and Sports of the Czech Republic project LO1417.

### Conflict of interest statement

The authors declare that the research was conducted in the absence of any commercial or financial relationships that could be construed as a potential conflict of interest.
